# Covid-19 and Capital Flows: The Responses of Investors to the Responses of Governments

**DOI:** 10.1007/s11079-021-09647-1

**Published:** 2022-02-28

**Authors:** Stefan Goldbach, Volker Nitsch

**Affiliations:** 1 Deutsche Bundesbank, Frankfurt, Germany; 2 Technische Universität Darmstadt, Darmstadt, Germany

**Keywords:** Coronavirus, pandemic, F36, F60, I18

## Abstract

This paper examines the effect of national government response measures to Covid-19 on German international capital flows. Analyzing highly disaggregated monthly data from the German balance of payments statistics over the period from January 2019 through January 2021, we find that bilateral financial interactions are negatively affected by stricter containment and closure policies as well as health system policies of a partner country, while German capital flows benefit from a partner’s economic support policies. Moreover, to the extent that public interventions to fight the pandemic affect financial interactions, the adjustment mainly takes place along the intensive margin.

## Introduction

The Covid-19 pandemic, and measures taken in response to the disease, dramatically affected economic behavior. Lacking an effective vaccine at that time, the Covid-19 outbreak triggered a massive rise in uncertainty (Baker et al. 2020). More notably, countries around the world implemented drastic measures, from border closures to national lockdowns, to slow and prevent the spread of the coronavirus.

The macroeconomic consequences of the pandemic have been dramatic. Economic activity temporarily collapsed on a global scale. According to the International Monetary Fund (2021), world output declined by more than 3 percent in 2020. Similarly, some cross-border activities, such as merchandise trade and travel, contracted sharply. For world trade, for instance, the World Trade Organization (2021) estimates a fall by more than 5 percent in 2020.

The impact of the pandemic on international capital flows, in contrast, is much less evident. At the beginning of the crisis, at a time of financial market turbulences, some countries experienced substantial capital outflows. In perspective, however, net outflows have been of a magnitude comparable to those seen in previous stress events (Batini 2020).^1^ Moreover, the flight-to-safety episode was remarkably short-lived, partly due to massive central bank intervention (Lane 2020).^2^

In principle, one can think of a number of channels through which a global pandemic might affect cross-border capital movements. One is the impact of uncertainty on international portfolio allocation. As risk increases, investors tend to reassess their positions, and portfolio realignment may then result in safe haven capital flows (Habib and Stracca 2013). Another channel focuses on the sensitivity of gross capital flows to economic conditions. Since capital flows are often found to be pro-cyclical, they are likely to fall during an economic slowdown, and maybe even to collapse in a crisis (Broner et al. 2013; Papaioannou et al. 2013). Moreover, measures to fight the pandemic, such as the closure of borders, may depress capital flows further.

In this paper, we examine empirically the effect of policy responses to the Covid-19 pandemic on German international capital flows. In particular, we make use of highly disaggregated monthly data from the German balance of payments statistics for the period from January 2019 to January 2021, which allows us to analyze the financial interactions of a single country, Germany, with foreign counterparts along multiple dimensions over the course of the pandemic. Exploiting then the variation in government response measures to Covid-19 across partner countries and time, we estimate the effect of national policies to slow the transmission of Covid-19 and economic support policies on a broad set of capital flow measures.^3^

Previewing our results, we find that German capital flows are sensitive to foreign policies in response to Covid-19, with the effect depending on the type of policies implemented. More specifically, German capital flows tend to decline when a partner country adopts stricter containment and closure policies. The stringency of lockdowns, therefore, has a negative effect on bilateral financial interactions, an effect which becomes larger the longer the pandemic drags on. For economic policies, in contrast, greater government intervention in response to Covid-19 seems to be associated with larger financial flows. Economic support measures tend to strengthen bilateral financial interactions, especially during the early phase of the pandemic. Finally, to the extent that public interventions to fight the pandemic affect financial interactions, the adjustment mainly takes place within relationships which continue to exist (i.e., along the intensive margin).

The remainder of the paper is organized as follows. In Section 2, we briefly discuss the relevant literature. Section 3 describes the data and our empirical strategy. Section 4 presents the empirical results. Finally, Section 5 briefly concludes.

## Literature

Our work is directly related to two strands of the literature. A first set of papers examines the determinants (or ‘drivers’) of bilateral capital flows. In this literature, often a gravity-type framework is applied to study patterns of cross-border financial holdings. While a wide range of ‘push’ and ‘pull’ factors for capital flows are identified, the analysis typically focuses on country-specific and global economic conditions; there is little evidence, if any, on the effect of health-related policy measures on capital flows. The literature is voluminous and has been extensively surveyed; see, among others, Montiel (2014) and Koepke (2019) for reviews.

Another line of research examines the impact of the Covid-19 global pandemic on economic activities, including cross-border interactions. A small, but rapidly-growing number of studies focuses on the effects of Covid-19 on international trade. Berthou and Stumpner (2021), for instance, use a large cross-country panel dataset and document a strong negative effect of lockdown policies on both exports and imports. Other studies analyze country-specific trade data and find similar results; examples include Benguria (2021) for Colombia and Amador et al. (2021) for Portugal. Still other studies explore the effects of the pandemic on non-trade cross-border flows, such as tourism; see, for instance, Cevik (2021).

Analyses of the response of capital flows to Covid-19, in contrast, have been mainly descriptive, often with a focus on the experiences of emerging market economies. The OECD (2020), for instance, provides a detailed discussion of capital flow dynamics during the pandemic and summarizes lessons from the history of sudden stops. Kalemli-Ozcan (2020) focuses on the composition of capital flows and discusses the implications of this composition during the shock episode, while, in another IMF publication, Batini (2020) reviews the policy responses to the capital flow volatility caused by the pandemic. A serious limitation is the (un)availability of detailed capital flows data at high frequencies; see ElFayoumi and Hengge (2021) for an exception.

By making use of granular information on Germany’s bilateral financial relationships with other countries, we are able to go beyond this work. Moreover, our focus on evidence from a single advanced economy, which may be considered a potential drawback, also has advantages, especially given that findings on the drivers of capital flows often differ along various dimensions, including the type of country, the type of capital flow and the asset category. In comparison with other countries, the pattern of Germany’s financial relationships can be characterized as diversified (across countries), balanced (between inflows and outflows) and stable (over time). Overall, our work complements country-specific studies on the impact of the Covid-19 pandemic on other cross-border activities, such as merchandise trade.

## Data and Methodology

In our analysis, we combine information from two datasets. Data on German cross-border financial activities are obtained from the Deutsche Bundesbank’s “Statistics on International Financial and Capital Transactions (SIFCT)”, which contains the microdata used for the compilation of the German balance of payments statistics.^4^ The data are provided at monthly frequency, based on mandatory reports by German declarants of financial transactions with foreign counterparts once they exceed a threshold value of 12,500 euro. Since each declaration contains numerous details, including the date and type of transaction, the partner country, the asset class and the value of the transactions, the number of individual entries is large. On average, more than 25,000 activities by about 4,000 declarants are recorded each month. In view of strict confidentiality restrictions, the micro data are only accessible, in anonymized form, at the headquarters of the Bundesbank in Frankfurt, Germany.

**Fig. 1:**
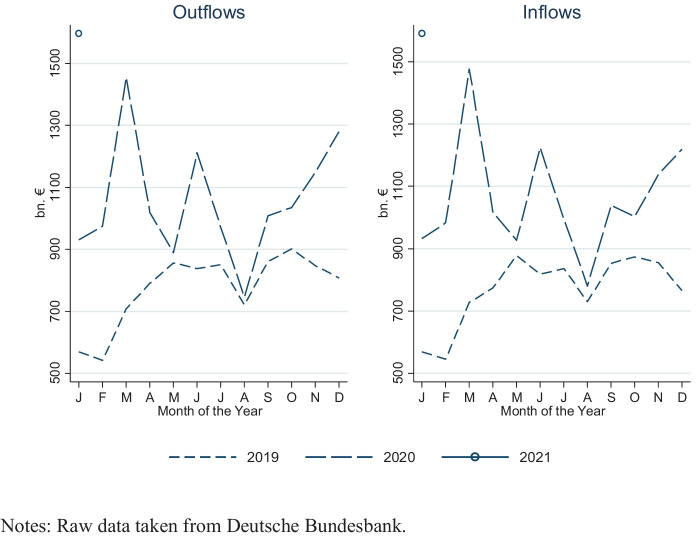
Monthly Capital Flows of Germany, 2019–21

Figure 1 plots the (aggregate) monthly capital flows into and out of Germany over time. Although the monthly figures are volatile (without a clear seasonal pattern), German capital flows have sizably increased on a year-over-year basis in 2020. This development, however, can, if anything, only partly be attributed to the pandemic. The strong correlation between inflows and outflows illustrates, for instance, the importance of bidirectional short-term capital movements (rather than, say, flight from risk assets activities). Another common factor, affecting both inflows and outflows simultaneously, is the United Kingdom’s withdrawal from the European Union (Brexit), with Britain-based banks moving parts of their international business to European Union countries, including Germany.^5^

We merge the German balance of payments data with information on how governments in the partner country respond to the Covid-19 pandemic. For our purposes of coding national coronavirus policies on a consistent basis, there are a number of data repositories available. We use data from the Oxford Covid-19 Government Response Tracker (OxCGRT).^6^ This database systematically collects publicly available information on policy measures that governments have taken to respond to the Covid-19 outbreak and reports 20 indicators of common government responses. These indicators are grouped into three broad categories: containment and closure policies (8 indicators), health system policies (8 indicators) and economic policies (4 indicators). In addition, the database contains various indices for which the data from the indicators is aggregated into a numerical value between 0 and 100.

The individual government response measures in the OxCGRT database are typically recorded on an ordinal scale. For ‘cancel public events’, for instance, the indicator takes integer values from 0 (no measures) to 2 (require cancelling), while for ‘restrictions on gatherings’ the indicator ranges from 0 (no restrictions) to 4 (restrictions on gatherings of 10 people or less). Only four of the 20 indicators are reported on a metric (US dollars) scale. In our empirical implementation, we use these indicators, despite the somewhat arbitrary definition of the ordinal measures, to allow for different intensities of the government responses.^7^ However, we transform the raw OxCGRT data, which is recorded at daily frequency, into monthly (arithmetic) averages for ordinal measures and into sums for the metric indicators which we can merge with our balance of payments data.^8^

**Fig. 2:**
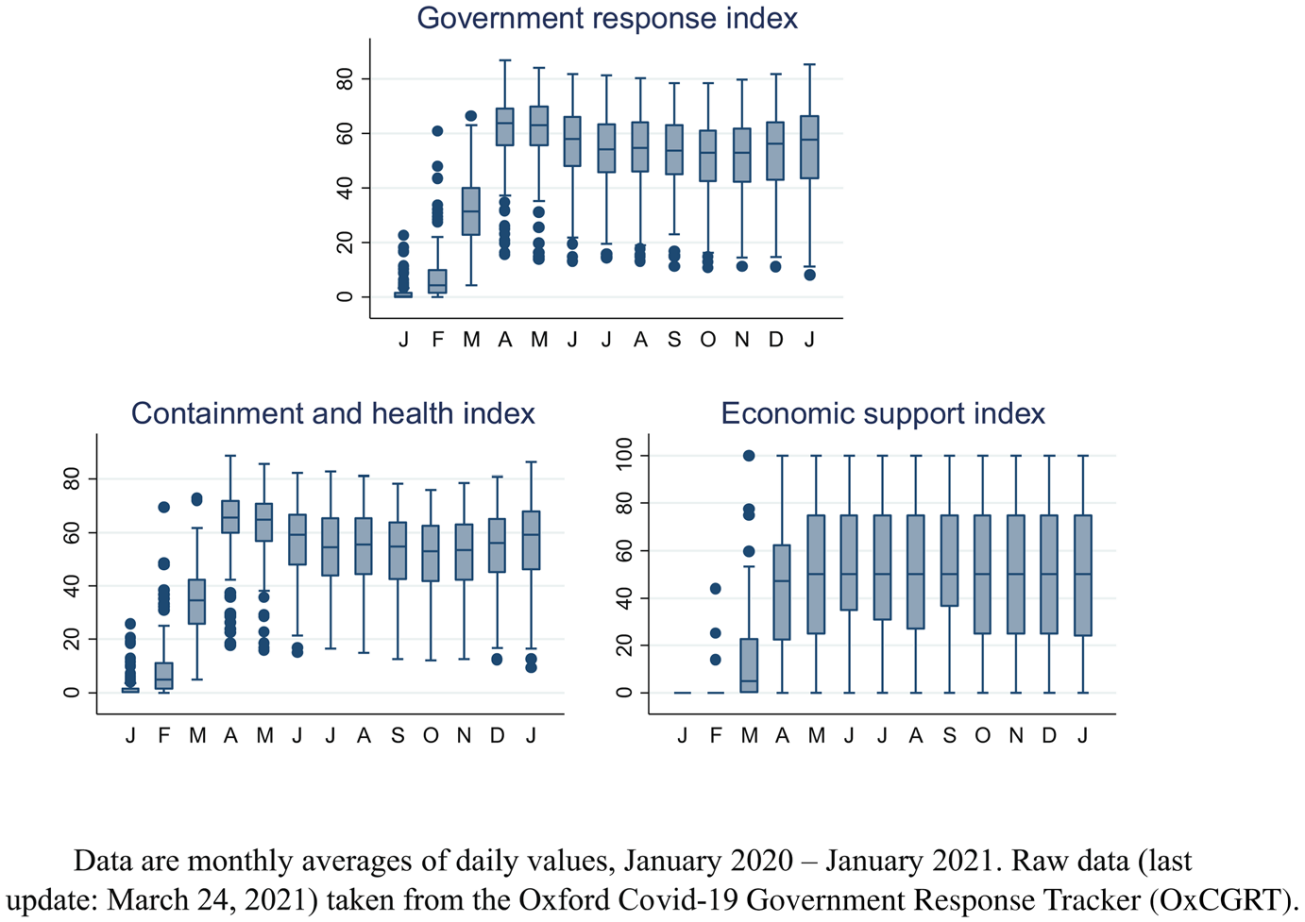
Composite Indicators of Government Response Measures to Covid-19

**Fig. 3:**
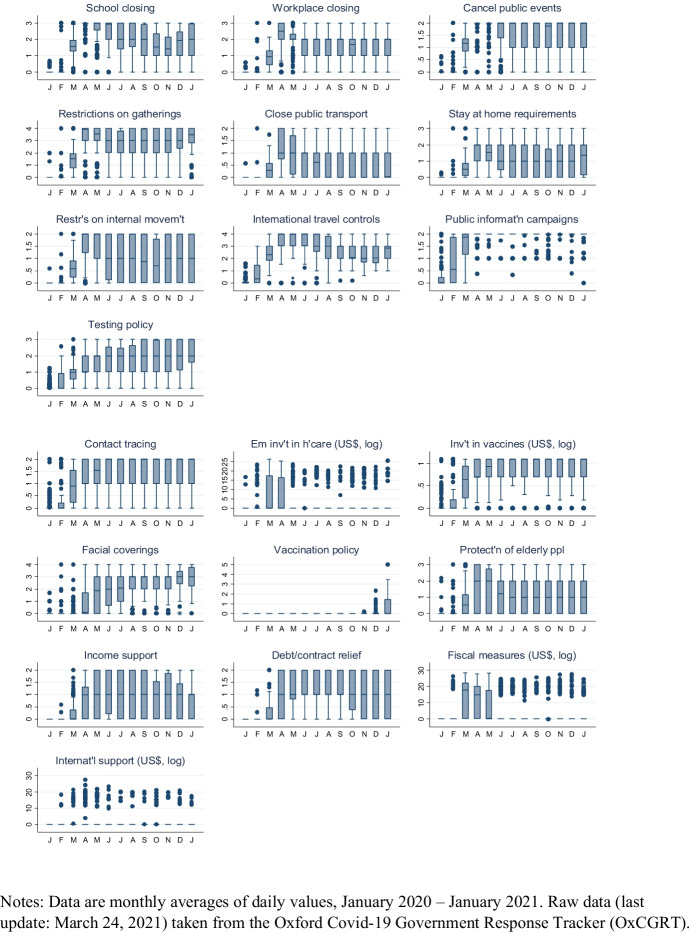
Government Response Measures to Covid-19

Figures 2 and 3 present monthly box plots of the OxCGRT indicators of government responses to Covid-19 across the 186 countries in the sample. The upper panel of Fig. 2 contains a box plot of the overall government response index, which summarizes the information from all (16) ordinal government response measures in a single index for each country; the two box plots in the lower panel present analogous composite indices for two types of government policies, effectively sorting each ordinal government response measure into one of two categories. Box plots for individual measures are presented in Fig. 3. Unsurprisingly, there is, in line with the spread of the coronavirus, a general tendency towards more and tighter government measures over the course of the year. However, many box plots are wide, indicating considerable cross-country variation in the implementation of these measures; there is also variation in when measures are adopted and (eventually) abandoned. For instance, many lockdown policies, such as school closures, have been temporary and lifted quickly as the number of Covid-19 cases declined, while other interventions, such as public information campaigns, are still widely in place. Overall, the box plots indicate considerable dispersion in government responses to Covid-19, both across countries and over time.^9^

In our empirical analysis, we make use of this variation to identify the impact of pandemic-related government interventions on cross-border financial flows. In particular, we compare the evolution of German financial activities with countries with high exposure to a government policy measure in response to Covid-19, relative to countries with low exposure to this measure. We do so by using a standard panel data model with country- and time-specific fixed effects, such that our basic empirical specification takes the form:

1 $$FinancialActiv{ity}_{ct}\hspace{0.17em}=\hspace{0.17em}C{ountry}_{c}\hspace{0.17em}+\hspace{0.17em}Mon{th}_{t}\hspace{0.17em}+\hspace{0.17em}\gamma GovtRespon{se}_{ct}\hspace{0.17em}\times \hspace{0.17em}Pandemic_{t}\hspace{0.17em}+\hspace{0.17em}\delta Contro{ls}_{ct}\hspace{0.17em}+\hspace{0.17em}{\varepsilon }_{ct},$$

where FinancialActivity_ct_ is a measure of German financial activity with country c in month t, Country_c_ and Month_t_ are comprehensive sets of country and time fixed effects, respectively, GovtResponse_ct_ is a measure of country c’s response to Covid-19 in month t, Pandemic_t_ is a binary dummy variable that takes the value of one for the period from January 2020 onwards (and is zero otherwise), Controls is a vector of additional controls, and ε is the residual. Following standard practice, our baseline measure of bilateral financial activity is the total value of capital flows (i.e., the sum of outflows and inflows).^10^ However, we also explore other indicators of a financial relationship (such as the number of declarants) as regressands.

In this conventional difference-in-differences setting, time fixed effects control for any variation in German financial flows that is common across all partners (including, for instance, fluctuations in external flows due the impact of Covid-19 in Germany), while country fixed effects absorb all factors that shape Germany’s financial relationship with a partner country and do not vary over time. By choosing a relatively short sample period then, our empirical set-up can be considered to be particularly demanding. At the same time, however, with this short time frame, it is difficult to identify factors which vary across countries and over time and significantly determine bilateral financial flows at monthly frequency. While we experiment with a large number of monetary and real variables,^11^ none of these additional controls consistently enters our equation statistically significantly. Moreover, to the extent these extensions affect the estimation results for our variable of interest, a foreign country’s government response to Covid-19, this effect is largely driven by changes in the size and composition of the sample (since the control variables are often only available for a subset of countries or with a time lag). Still, for illustration, we report results for augmented versions of Eq. (1) in which we control for a foreign country’s economic outlook by using the monthly percentage change in the national central bank’s balance sheet and the OECD composite leading indicator.^12^^,^ 13

## Results

We begin our empirical analysis by estimating Eq. (1) at the most aggregate level, using the OxCGRT summary indicators to quantify the government responses to Covid-19 in countries abroad. Table 1 reports the results. Our default estimate is recorded in the first column of the upper panel of the table. In this column, we tabulate the regression results from the most parsimonious specification of Eq. (1), which only includes the variable of interest (along with country and time fixed effects), for the overall government response index. The estimate of γ is small and not significantly different from zero at any confidence level, indicating that German capital flows are insensitive to policies implemented abroad to fight the pandemic. Moreover, this (non-)finding turns out to be robust when we sequentially include, at the cost of a substantially reduced sample size, additional regressors to capture the effects of a partner country’s monetary and macroeconomic conditions on capital flows. As shown in columns (2) to (4), the γ coefficient remains insignificant in these extended specifications (but increases in magnitude). For a composite indicator of government responses to Covid-19, therefore, which aggregates the information from 16 different indicators of government measures into a single index, there is no evidence that the pattern of bilateral capital flows is sizably affected by policy measures taken in response to Covid-19.

In the remaining eight columns of the table, we present sets of analogous results for two sub-indices which summarize information on different types of public policies in response to Covid-19. Columns (5) to (8) show the results when the overall government response index is replaced with a summary index of containment and health policies only; this index aggregates the information from 14 (of the 16) indicators in the overall index.^14^ For this measure, then, the estimated effect on capital flows is not only consistently negative; the effect is also marginally significant at the 10 percent level in two of the four specifications. Consequently, bilateral financial relationships do not seem to be completely unaffected by the pandemic. At least for the group of Germany’s major financial partners (for most of which data on the composite leading indicator is available), capital flows fall sizably when a country implements stricter lockdown policies. While we do not attempt to interpret the point estimates too literally, given the arbitrary construction of the index, a 10 percentage point increase in the containment and health index is associated with a 5 percent decrease in bilateral capital flows.

For a summary index of economic policies, in contrast, which aggregates the information from the two remaining ordinal indicators (that is, indicators dealing with economic policy measures in response to Covid-19), the estimate of γ is always positive and, with one exception, statistically significant at conventional levels. According to these findings, financial relationships benefit from more active policies abroad, policies with which governments provide economic support to reduce the impact of the pandemic. Moreover, it is reassuring to note that the results are remarkably robust, holding for both the worldwide sample of 160 countries and for specifications when we additionally control for a foreign country’s economic outlook and, thereby, effectively reduce the sample to Germany’s main partners for financial business.

Next, instead of estimating the average effect over the course of the pandemic, we allow for two separate interaction terms which distinguish between the first (before September 2020) and the second wave (from September 2020 onwards) of the pandemic.^15^ The results are presented in the lower panel of Table 1, which tabulates estimates for specifications of Eq. (1) analogous to those that have been reported in the upper panel of the table. Again, the effects of government response measures to Covid-19 on capital flows vary strongly by type of public policies, although many coefficients lack statistical significance at this aggregate level. For containment and health policies, the estimates suggest that stricter measures tend to reduce financial interactions, and this (negative) effect seems to become larger over time. For economic support policies, in contrast, a more active policy stance benefits cross-border financial transactions, with particularly strong effects immediately after the outbreak of the pandemic. One obvious and plausible explanation of this pattern is that the economic costs of Covid-19 increase with the duration of the pandemic.

In Table 2, we repeat the regression analysis, using the individual OxCGRT indicators of government response measures in place of the composite indices. As before, the upper panel reports the average effect, where we limit the analysis to the least (baseline) and most demanding (with additional controls) specifications of Eq. (1) in Table 1; the lower panel reports the analogues when we again allow the effect to differ by wave. In total, the table presents the results of (20 × 4 =) 80 regressions. However, to save space, we tabulate only the estimated coefficients of interest.

Reviewing the results, the estimates turn out to be generally in line with our initial findings. German capital flows rarely respond in systematic ways to actions taken by foreign governments in response to the pandemic; the majority of the estimates is not significantly different from zero. For some government responses, such as ‘public information campaigns’, for instance, none of the specifications provides an estimate of γ which is significant at any level. More importantly, to the extent that an effect of government measures on bilateral financial relationships is identifiable, the results strongly confirm our findings for the composite indices. Hard lockdown policies lead to a reduction in financial interactions, especially the longer the pandemic drags on; also, it seems plausible that the strongest (negative) effects are obtained for ‘workplace closings’. Public interventions providing economic support, in contrast, tend to benefit financial relationships; of the four indicators that track a country’s economic policies, especially the variable recording direct ‘income support’ measures for households enters (mostly) significantly and with a positive sign, with possible explanations including household and firm access to financial resources available for cross-border transfer and expectations of an economy better being able to cope with the economic consequences of the pandemic-induced crisis.^16^

In a further disaggregation, we analyze capital inflows and capital outflows separately. In particular, it may be argued that the pandemic, and national policy measures taken in response to the crisis, affect different types of capital flows differently. Stricter lockdown policies, for instance, may increase a country’s capital flows to Germany, while (expansionary) economic support policies may particularly benefit capital flows to a country from Germany (e.g., due to expectations of an improvement in the economic outlook and the performance of a country’s domestic financial markets). Therefore, to analyze this issue, Tables 3a and 3b provide analogues to Table 2 for capital inflows and capital outflows, respectively.

Interestingly, the estimates turn out to be remarkably robust, both in terms of magnitude and statistical significance, irrespective of the direction of the capital flow. This finding may be specific to Germany as an advanced country, in contrast to emerging market and developing economies. Still, the similarity in the response patterns of capital flows indicates that the pandemic affects Germany’s bilateral financial relationships rather than unidirectional flows to and from Germany.

We have performed extensive robustness checks to further analyze the sensitivity of our results along various lines. In one exercise, for instance, we analyze a subsample comprising European Union member countries only. We also exclude countries serving as international financial center. None of these perturbations, measurably affects our results.

In a next step, we explore possible channels of the effect of pandemic-related public interventions on capital flows. To examine bilateral financial relationships in more detail, we make use of the granular nature of our financial flows data. We begin by analyzing financial flows by asset category.

Table 4 presents the results. In this table, we report separate estimates for financial activities in six different asset categories.^17^ However, to avoid unnecessary cluttering, we only tabulate the results for two selected government measures in response to Covid-19, ‘workplace closing’ and ‘income support’; for these indicators, out of the set of 20 indicators compiled by OxCGRT, we find the most consistent and strongest (oppositely signed) effects on capital flows.^18^ Thus the first six columns of the table provide the results for ‘workplace closing’; each of these columns contains the coefficient estimates for capital flows in one of the six different asset categories (bonds, equities, etc.). The remaining six columns present the analogues for ‘income support’. Since we again report the results for four different specifications of Eq. (1), the table contains, in total, the results of (12 × 4 =) 48 regressions.

Without placing too much emphasis on individual estimates, an interesting pattern is observable. For one thing, there is, perhaps not surprisingly, considerable heterogeneity in the estimated effects across different asset categories. More notably, the policy measures particularly affect financial activities in bonds, equities and money market instruments which are not only the largest but also the most liquid asset categories. As a result, pandemic-related policy interventions have wide-ranging consequences for cross-border financial interactions of all types. Overall, we consider this finding generally reassuring.

In another exercise, we decompose the effect of public interventions in response to Covid-19 on capital flows along extensive and intensive margins. In particular, we decompose the aggregate value of German capital flows with a partner country into various components, including the unique number of reporting units that declare financial transactions with that country, the unique number of asset classes in which business has taken place, and the average value of capital flows by declarant-asset pair. By substituting then these measures, in place of the default total value, as dependent variable, we are able to identify the contributions of these components to the overall effect.

The results are presented in Table 5. Again, we focus, for brevity, on the effects of a policy measure to contain the spread of Covid-19, ‘workplace closing’, and a policy measure that aims to provide support to better cope with the economic consequences of the pandemic, ‘income support’. Consequently, the columns (1) and (7) replicate, for comparison, the corresponding estimates from Table 2, while the remaining columns tabulate the analogous estimation results for the margins recorded at the top of the column. As before, the estimates of γ vary substantially, in both economic and statistical terms, but for each variable of interest (i.e., margin), the results turn out to be remarkably robust across different specifications. In particular, it is interesting to note that public interventions to fight the Covid-19 pandemic predominantly affect capital flows through the intensive margin. While average values of transactions adjust, the scope of financial activities remains largely unaffected; that is, the pandemic-related financial adjustments mainly take place within relationships which continue to exist. These results are in contrast to findings for other types of policy interventions such as, for instance, financial sanctions (Besedeš et al. 2021).

## Conclusions

Since the beginning of 2020, cross-border economic activities are shaped by a global pandemic. Aiming to slow the spread of the coronavirus, governments have taken drastic interventions, with serious economic consequences. Most notably, various types of cross-border interactions (such as international travel) were heavily restricted or banned completely.

In this paper, we examine the effect of national government response measures to the pandemic on international capital flows. Based on an analysis of highly disaggregated monthly data from the German balance of payments statistics over the period from January 2019 through January 2021, we find that bilateral financial interactions are negatively affected by stricter lockdown policies abroad, especially the longer the pandemic lasts. Economic support policies, in contrast, tend to benefit capital flows, with the largest effects during the initial phase of the pandemic. We conclude that cross-border capital flows are sensitive to policies in response to Covid-19. While considerable heterogeneity in the estimated effects across different asset categories exists, the policy measures particularly affect financial activities in bonds, equities and money market instruments which are not only the largest but also the most liquid asset categories.

**Table 1:** The Effect of Aggregate Government Response Measures to Covid-19 on German Capital Flows

	Government response index (index, 0–1)				Containment and health index (index, 0–1)				Economic support index (index, 0–1)			
a) Total effect	(1)	(2)	(3)	(4)	(5)	(6)	(7)	(8)	(9)	(10)	(11)	(12)
Response measure × Covid-19 pandemic	-0.046 (0.391)	-0.275 (0.352)	-0.533 (0.397)	-0.630 (0.430)	-0.246 (0.396)	-0.422 (0.310)	-0.666# (0.368)	-0.775# (0.415)	0.415* (0.151)	0.260 (0.172)	0.263# (0.138)	0.248# (0.142)
Central bank balance sheet (change, m/m)		1.226** (0.381)		0.204 (0.243)		1.228** (0.383)		0.190 (0.239)		1.209** (0.382)		0.207 (0.241)
Composite leading indicator (normalized)			-2.684 (1.631)	-4.645* (2.146)			-2.802# (1.590)	-4.611* (2.138)			-1.655 (1.594)	-3.849 (2.270)
# Countries	160	132	38	36	160	132	38	36	160	132	38	36
# Observations	4,000	2,795	927	860	4,000	2,795	927	860	4,000	2,795	927	860
Adj. R^2^	0.932	0.946	0.974	0.976	0.932	0.946	0.974	0.976	0.932	0.946	0.974	0.975
b) Effect by Covid-19 wave	(1)	(2)	(3)	(4)	(5)	(6)	(7)	(8)	(9)	(10)	(11)	(12)
Response measure × First wave Covid-19	0.213 (0.415)	-0.663# (0.356)	-0.515 (0.415)	-0.595 (0.483)	0.040 (0.434)	-0.731* (0.315)	-0.622 (0.397)	-0.715 (0.477)	0.446* (0.166)	0.150 (0.156)	0.292* (0.106)	0.272* (0.120)
Response measure × Second wave Covid-19	-0.406 (0.530)	0.330 (0.296)	-0.562 (0.531)	-0.680 (0.507)	-0.660 (0.548)	0.121 (0.312)	-0.749 (0.457)	-0.882# (0.453)	0.381# (0.187)	0.425# (0.247)	0.222 (0.244)	0.212 (0.253)
Central bank balance sheet (change, m/m)		1.239** (0.378)		0.204 (0.243)		1.234** (0.381)		0.191 (0.239)		1.223** (0.377)		0.202 (0.242)
Composite leading indicator (normalized)			-2.662 (1.707)	-4.634* (2.157)			-2.749 (1.648)	-4.607* (2.143)			-1.614 (1.598)	-3.804 (2.296)
# Countries	160	132	38	36	160	132	38	36	160	132	38	36
# Observations	4,000	2,795	927	860	4,000	2,795	927	860	4,000	2,795	927	860
Adj. R^2^	0.932	0.946	0.974	0.976	0.932	0.946	0.974	0.976	0.932	0.946	0.974	0.975

OLS estimation. The dependent variable is the log of bilateral capital flows of Germany with other countries. The unit of observation is a country-month pair. Data cover the period from January 2019 through January 2021 in monthly frequency. Time fixed effects and country fixed effects are included but not reported. Robust standard errors (clustered by time and country) are in parentheses. **, * and # denote significant at the 1%, 5% and 10% level, respectively.

**Table 2:** The Effect of Government Response Measures to Covid-19 on German Capital Flows

	Containment and closure policies								Health system policies	
	School closing (index, 0–3)	Workplace closing (index, 0–3)	Cancel public events (index, 0–2)	Restrictions on gatherings (index, 0–4)	Close public transport (index, 0–2)	Stay at home requirements (index, 0–3)	Restrictions on internal movement (index, 0–2)	International travel controls (index, 0–4)	Public information campaigns (index, 0–2)	Testing policy (index, 0–3)
a) Total effect	(1)	(2)	(3)	(4)	(5)	(6)	(7)	(8)	(9)	(10)
	Baseline									
Response measure × Covid-19 pandemic	-0.050 (0.069)	-0.122* (0.058)	-0.059 (0.079)	-0.031 (0.051)	-0.126# (0.071)	-0.166* (0.075)	-0.205* (0.078)	-0.039 (0.052)	0.045 (0.088)	0.213** (0.069)
	With additional controls									
Response measure × Covid-19 pandemic	-0.083 (0.050)	-0.149** (0.043)	-0.136* (0.065)	-0.026 (0.026)	-0.050 (0.077)	-0.119* (0.053)	-0.093# (0.051)	0.032 (0.035)	-0.004 (0.050)	-0.020 (0.052)
b) Effect by Covid-19 wave	(1)	(2)	(3)	(4)	(5)	(6)	(7)	(8)	(9)	(10)
	Baseline									
Response measure × First wave Covid-19	0.010 (0.087)	-0.085 (0.088)	-0.008 (0.118)	-0.012 (0.063)	-0.070 (0.081)	-0.111 (0.088)	-0.127 (0.110)	0.054 (0.061)	0.114 (0.085)	0.182* (0.073)
Response measure × Second wave Covid-19	-0.118 (0.097)	-0.168** (0.046)	-0.111 (0.093)	-0.051 (0.072)	-0.213 (0.134)	-0.228* (0.082)	-0.277** (0.093)	-0.180** (0.050)	-0.172 (0.161)	0.250** (0.079)
	With additional controls									
Response measure × First wave Covid-19	-0.053 (0.064)	-0.123* (0.048)	-0.115 (0.072)	-0.020 (0.031)	-0.042 (0.087)	-0.107# (0.062)	-0.084 (0.064)	0.002 (0.042)	-0.004 (0.050)	-0.050 (0.045)
Response measure × Second wave Covid-19	-0.130* (0.059)	-0.189** (0.058)	-0.168* (0.062)	-0.040 (0.037)	-0.062 (0.098)	-0.137# (0.078)	-0.101 (0.070)	0.105# (0.054)	0.000 (0.000)	0.057 (0.083)

	Health system policies						Economic support policies			
	Contact tracing (index, 0–2)	Emergency inv’t in healthcare (USD, ln)	Investment in vaccines (USD, ln)	Facial coverings (index, 0–4)	Vaccination policy (index, 0–5)	Protection of elderly people (index, 0–3)	Income support (index, 0–2)	Debt/contract relief (index, 0–2)	Fiscal measures (USD, ln)	International support (USD, ln)
a) Total effect	(11)	(12)	(13)	(14)	(15)	(16)	(17)	(18)	(19)	(20)
	Baseline									
Response measure × Covid-19 pandemic	0.177# (0.095)	-0.035# (0.020)	0.020 (0.013)	0.048 (0.045)	0.272** (0.040)	0.036 (0.053)	0.259** (0.066)	0.034 (0.065)	-0.011 (0.011)	-0.020 (0.020)
	With additional controls									
Response measure × Covid-19 pandemic	-0.105# (0.061)	-0.016 (0.011)	-0.105# (0.061)	-0.016 (0.011)	0.067 (0.067)	-0.036 (0.036)	0.080 (0.080)	0.078 (0.051)	0.000 (0.004)	0.004 (0.010)
b) Effect by Covid-19 wave	(11)	(12)	(13)	(14)	(15)	(16)	(17)	(18)	(19)	(20)
	Baseline									
Response measure × First wave Covid-19	0.134 (0.111)	-0.027 (0.020)	0.010 (0.017)	0.047 (0.053)	–	0.024 (0.055)	0.243** (0.071)	0.072 (0.067)	-0.018 (0.014)	-0.030 (0.024)
Response measure × Second wave Covid-19	0.249* (0.115)	-0.066* (0.031)	0.032# (0.019)	0.051 (0.060)	0.272** (0.040)	0.049 (0.069)	0.276** (0.080)	-0.008 (0.075)	0.011 (0.022)	0.009 (0.026)
	With additional controls									
Response measure × First wave Covid-19	-0.092 (0.058)	-0.007 (0.009)	0.007 (0.013)	-0.057 (0.043)	–	-0.029 (0.035)	0.129# (0.071)	0.056 (0.049)	-0.003 (0.005)	0.003 (0.010)
Response measure × Second wave Covid-19	-0.149 (0.094)	-0.039** (0.013)	0.026# (0.013)	-0.101* (0.046)	0.067 (0.067)	-0.046 (0.048)	0.001 (0.113)	0.110 (0.073)	0.006 (0.008)	0.008 (0.015)

OLS estimation. The dependent variable is the log of bilateral capital flows of Germany with other countries. The unit of observation is a country-month pair. Data cover the period from January 2019 through January 2021 in monthly frequency. Time fixed effects and country fixed effects are included but not reported. Robust standard errors (clustered by time and country) are in parentheses. **, * and # denote significant at the 1%, 5% and 10% level, respectively.

**Table 3:** a: The Effect of Government Response Measures to Covid-19 on German Capital Inflows

	Containment and closure policies																Health system policies	
	School closing (index, 0–3)		Workplace closing (index, 0–3)		Cancel public events (index, 0–2)		Restrictions on gatherings (index, 0–4)		Close public transport (index, 0–2)		Stay at home requirements (index, 0–3)		Restrictions on internal movement (index, 0–2)		International travel controls (index, 0–4)		Public information campaigns (index, 0–2)	Testing policy (index, 0–3)
a) Total effect	(1)		(2)		(3)		(4)		(5)		(6)		(7)		(8)		(9)	(10)
	Baseline																	
Response measure × Covid-19 pandemic	-0.053 (0.052)		-0.066 (0.048)		-0.068 (0.066)		-0.037 (0.035)		-0.114# (0.065)		-0.114# (0.063)		-0.171* (0.065)		-0.040 (0.042)		-0.014 (0.083)	0.136* (0.064)
	With additional controls																	
Response measure × Covid-19 pandemic	-0.081 (0.052)		-0.147** (0.046)		-0.155* (0.074)		-0.027 (0.024)		-0.047 (0.073)		-0.112# (0.058)		-0.076 (0.053)		0.019 (0.037)		0.001 (0.042)	-0.034 (0.051)
b) Effect by Covid-19 wave	(1)		(2)		(3)		(4)		(5)		(6)		(7)		(8)		(9)	(10)
	Baseline																	
Response measure × First wave Covid-19	-0.031 (0.062)		-0.030 (0.054)		-0.067 (0.085)		-0.034 (0.043)		-0.070 (0.072)		-0.094 (0.079)		-0.116 (0.073)		0.005 (0.049)		0.007 (0.088)	0.070 (0.072)
Response measure × Second wave Covid-19	-0.078 (0.076)		-0.112# (0.060)		-0.069 (0.084)		-0.040 (0.050)		-0.180 (0.117)		-0.136# (0.073)		-0.222* (0.084)		-0.107* (0.046)		-0.080 (0.164)	0.214** (0.077)
	With additional controls																	
Response measure × First wave Covid-19	-0.057 (0.065)		-0.130* (0.050)		-0.140# (0.074)		-0.025 (0.031)		-0.046 (0.081)		-0.113# (0.066)		-0.069 (0.068)		-0.018 (0.045)		0.001 (0.042)	-0.062 (0.043)
Response measure × Second wave Covid-19	-0.119# (0.064)		-0.173* (0.063)		-0.180* (0.075)		-0.032 (0.029)		-0.048 (0.095)		-0.111 (0.084)		-0.082 (0.070)		0.110* (0.050)		0.000 (0.000)	0.042 (0.091)

		Health system policies												Economic support policies				
		Contact tracing (index, 0–2)		Emergency inv’t in healthcare (USD, ln)		Investment in vaccines (USD, ln)		Facial coverings (index, 0–4)		Vaccination policy (index, 0–5)		Protection of elderly people (index, 0–3)		Income support (index, 0–2)		Debt/contract relief (index, 0–2)	Fiscal measures (USD, ln)	International support (USD, ln)
a) Total effect		(11)		(12)		(13)		(14)		(15)		(16)		(17)		(18)	(19)	(20)
		Baseline																
Response measure × Covid-19 pandemic		0.127 (0.084)		-0.023 (0.015)		0.018 (0.011)		-0.010 (0.043)		0.316** (0.039)		0.060 (0.051)		0.295** (0.060)		-0.017 (0.075)	0.001 (0.009)	-0.011 (0.021)
		With additional controls																
Response measure × Covid-19 pandemic		-0.118 (0.078)		-0.019 (0.012)		0.009 (0.008)		-0.070 (0.039)		0.055 (0.073)		-0.039 (0.035)		0.102 (0.085)		0.092# (0.054)	-0.001 (0.004)	0.005 (0.010)
b) Effect by Covid-19 wave		(11)		(12)		(13)		(14)		(15)		(16)		(17)		(18)	(19)	(20)
		Baseline																
Response measure × First wave Covid-19		0.089 (0.100)		-0.011 (0.015)		0.011 (0.017)		-0.011 (0.047)		–		0.058 (0.059)		0.301** (0.069)		-0.031 (0.074)	-0.003 (0.010)	-0.018 (0.026)
Response measure × Second wave Covid-19		0.192 (0.116)		-0.065* (0.029)		0.027# (0.016)		-0.007 (0.054)		0.316** (0.039)		0.062 (0.061)		0.289** (0.069)		-0.001 (0.103)	0.013 (0.015)	0.010 (0.028)
		With additional controls																
Response measure × First wave Covid-19		-0.113 (0.077)		-0.007 (0.008)		-0.002 (0.012)		-0.063 (0.041)		–		-0.032 (0.036)		0.148* (0.066)		0.052 (0.051)	-0.003 (0.005)	0.003 (0.010)
Response measure × Second wave Covid-19		-0.135 (0.097)		-0.049* (0.018)		0.025* (0.011)		-0.083# (0.047)		0.055 (0.073)		-0.050 (0.048)		0.029 (0.122)		0.148# (0.072)	0.006 (0.009)	0.011 (0.014)

OLS estimation. The dependent variable is the log of bilateral capital inflows of Germany with other countries. The unit of observation is a country-month pair. Data cover the period from January 2019 through January 2021 in monthly frequency. Time fixed effects and country fixed effects are included but not reported. Robust standard errors (clustered by time and country) are in parentheses. **, * and # denote significant at the 1%, 5% and 10% level, respectively.

**Table 3:** b: The Effect of Government Response Measures to Covid-19 on German Capital Outflows

	Containment and closure policies								Health system policies	
	School closing (index, 0–3)	Workplace closing (index, 0–3)	Cancel public events (index, 0–2)	Restrictions on gatherings (index, 0–4)	Close public transport (index, 0–2)	Stay at home requirements (index, 0–3)	Restrictions on internal movement (index, 0–2)	International travel controls (index, 0–4)	Public information campaigns (index, 0–2)	Testing policy (index, 0–3)
a) Total effect	(1)	(2)	(3)	(4)	(5)	(6)	(7)	(8)	(9)	(10)
	Baseline									
Response measure × Covid-19 pandemic	-0.046 (0.057)	-0.125* (0.056)	-0.050 (0.067)	-0.032 (0.046)	-0.078 (0.057)	-0.185** (0.064)	-0.177* (0.072)	-0.013 (0.039)	0.075 (0.076)	0.193** (0.064)
	With additional controls									
Response measure × Covid-19 pandemic	-0.089# (0.048)	-0.145** (0.043)	-0.115# (0.060)	-0.023 (0.028)	-0.044 (0.081)	-0.118* (0.050)	-0.105# (0.052)	0.038 (0.036)	-0.002 (0.063)	-0.011 (0.053)
b) Effect by Covid-19 wave	(1)	(2)	(3)	(4)	(5)	(6)	(7)	(8)	(9)	(10)
	Baseline									
Response measure × First wave Covid-19	-0.037 (0.076)	-0.109 (0.094)	-0.028 (0.119)	-0.032 (0.063)	-0.043 (0.074)	-0.143# (0.076)	-0.162 (0.113)	0.052 (0.049)	0.097 (0.083)	0.181* (0.070)
Response measure × Second wave Covid-19	-0.055 (0.082)	-0.144** (0.039)	-0.073 (0.077)	-0.031 (0.061)	-0.133 (0.091)	-0.232** (0.070)	-0.191* (0.076)	-0.112* (0.040)	0.008 (0.135)	0.206* (0.075)
	With additional controls									
Response measure × First wave Covid-19	-0.054 (0.060)	-0.106# (0.051)	-0.088 (0.071)	-0.012 (0.031)	-0.027 (0.090)	-0.092 (0.063)	-0.092 (0.067)	0.016 (0.042)	-0.002 (0.063)	-0.039 (0.047)
Response measure × Second wave Covid-19	-0.142* (0.056)	-0.205** (0.049)	-0.158* (0.058)	-0.050 (0.043)	-0.068 (0.107)	-0.156* (0.071)	-0.118 (0.073)	0.091 (0.058)	0.000 (0.000)	0.060 (0.081)

	Health system policies						Economic support policies			
	Contact tracing (index, 0–2)	Emergency inv’t in healthcare (USD, ln)	Investment in vaccines (USD, ln)	Facial coverings (index, 0–4)	Vaccination policy (index, 0–5)	Protection of elderly people (index, 0–3)	Income support (index, 0–2)	Debt/contract relief (index, 0–2)	Fiscal measures (USD, ln)	International support (USD, ln)
a) Total effect	(11)	(12)	(13)	(14)	(15)	(16)	(17)	(18)	(19)	(20)
	Baseline									
Response measure × Covid-19 pandemic	0.189* (0.085)	-0.021 (0.022)	0.031# (0.015)	0.045 (0.048)	0.185** (0.040)	0.021 (0.052)	0.216** (0.068)	0.048 (0.062)	0.003 (0.011)	0.003 (0.015)
	With additional controls									
Response measure × Covid-19 pandemic	-0.100# (0.058)	-0.011 (0.009)	0.018 (0.011)	-0.073# (0.040)	0.061 (0.059)	-0.033 (0.037)	0.045 (0.078)	0.068 (0.049)	0.001 (0.005)	0.002 (0.009)
b) Effect by Covid-19 wave	(11)	(12)	(13)	(14)	(15)	(16)	(17)	(18)	(19)	(20)
	Baseline									
Response measure × First wave Covid-19	0.131 (0.090)	-0.015 (0.026)	0.030 (0.019)	0.016 (0.059)	–	0.016 (0.053)	0.222* (0.081)	0.062 (0.073)	-0.001 (0.013)	0.007 (0.018)
Response measure × Second wave Covid-19	0.286* (0.104)	-0.042# (0.024)	0.033# (0.019)	0.094# (0.053)	0.185** (0.040)	0.027 (0.066)	0.210* (0.079)	0.032 (0.075)	0.015 (0.025)	-0.009 (0.022)
	With additional controls									
Response measure × First wave Covid-19	-0.083 (0.054)	-0.004 (0.010)	0.013 (0.015)	-0.050 (0.043)	–	-0.026 (0.035)	0.099 (0.078)	0.061 (0.046)	-0.001 (0.006)	0.001 (0.010)
Response measure × Second wave Covid-19	-0.157 (0.098)	-0.029* (0.011)	0.026 (0.016)	-0.114* (0.047)	0.061 (0.059)	-0.044 (0.052)	-0.042 (0.106)	0.078 (0.075)	0.005 (0.008)	0.004 (0.015)

OLS estimation. The dependent variable is the log of bilateral capital outflows of Germany with other countries. The unit of observation is a country-month pair. Data cover the period from January 2019 through January 2021 in monthly frequency. Time fixed effects and country fixed effects are included but not reported. Robust standard errors (clustered by time and country) are in parentheses. **, * and # denote significant at the 1%, 5% and 10% level, respectively.

**Table 4:** The Effect of Selected Government Response Measures on Capital Flows by Asset Category

	Workplace closing						Income support					
	Bonds	Money market instruments	Equity	Investment certificates	Foreign direct investment	Other	Bonds	Money market instruments	Equity	Investment certificates	Foreign direct investment	Other
a) Total effect	(1)	(2)	(3)	(4)	(5)	(6)	(7)	(8)	(9)	(10)	(11)	(12)
	Baseline											
Response measure × Covid-19 pandemic	0.155# (0.076)	0.263 (0.173)	0.087 (0.065)	0.142# (0.079)	0.235* (0.112)	0.156 (0.122)	0.317** (0.086)	0.499** (0.150)	0.199* (0.081)	0.127 (0.097)	0.309* (0.137)	0.182 (0.119)
	With additional controls											
Response measure × Covid-19 pandemic	-0.149* (0.062)	-0.029 (0.144)	-0.111* (0.043)	0.006 (0.190)	0.060 (0.087)	0.118 (0.117)	0.065 (0.095)	-0.387 (0.254)	0.042 (0.052)	-0.218 (0.132)	-0.207# (0.113)	0.157* (0.073)
b) Effect by Covid-19 wave	(1)	(2)	(3)	(4)	(5)	(6)	(7)	(8)	(9)	(10)	(11)	(12)
	Baseline											
Response measure × First wave Covid-19	0.232* (0.097)	0.299 (0.186)	0.127 (0.083)	0.175 (0.108)	0.295* (0.125)	0.259 (0.146)	0.345** (0.108)	0.593** (0.159)	0.231** (0.074)	0.166 (0.139)	0.265# (0.137)	0.334* (0.123)
Response measure × Second wave Covid-19	0.049 (0.053)	0.223 (0.189)	0.035 (0.064)	0.092 (0.078)	0.155 (0.139)	0.015 (0.112)	0.285** (0.100)	0.406* (0.160)	0.165# (0.095)	0.085 (0.098)	0.355# (0.185)	0.017 (0.120)
	With additional controls											
Response measure × First wave Covid-19	-0.135# (0.067)	0.146 (0.223)	-0.077 (0.054)	0.006 (0.188)	0.168# (0.083)	0.003 (0.132)	0.106* (0.050)	-0.461 (0.407)	0.114* (0.046)	-0.156 (0.155)	-0.297* (0.111)	0.054 (0.118)
Response measure × Second wave Covid-19	-0.171* (0.073)	-0.282** (0.086)	-0.166** (0.044)	0.007 (0.233)	-0.114 (0.108)	0.298# (0.155)	-0.001 (0.177)	-0.276 (0.456)	-0.073 (0.068)	-0.310# (0.159)	-0.056 (0.150)	0.320* (0.124)

OLS estimation. The dependent variable is the log of bilateral capital flows of Germany with other countries in the asset category recorded at the top of the column. The unit of observation is a country-month pair. Data cover the period from January 2019 through January 2021 in monthly frequency. Time fixed effects and country fixed effects are included but not reported. Robust standard errors (clustered by time and country) are in parentheses. **, * and # denote significant at the 1%, 5% and 10% level, respectively.

**Table 5:** The Effect of Selected Government Response Measures to Covid-19 on German Financial Activities

	Workplace closing						Income support					
	Log Total Value	Log Number of Entries	Log Avg. Value per Entry	Log Number of Declarants	Log Number of Asset Classes	Log Avg. Value per Asset Class per Declarant	Log Total Value	Log Number of Entries	Log Avg. Value per Entry	Log Number of Declarants	Log Number of Asset Classes	Log Avg. Value per Asset Class per Declarant
a) Total effect	(1)	(2)	(3)	(4)	(5)	(6)	(7)	(8)	(9)	(10)	(11)	(12)
	Baseline											
Response measure × Covid-19 pandemic	-0.122* (0.058)	-0.012 (0.012)	-0.110* (0.051)	-0.015 (0.011)	-0.006 (0.007)	-0.101# (0.054)	0.259** (0.066)	0.028 (0.021)	0.232** (0.053)	0.017 (0.019)	0.018 (0.011)	0.225** (0.051)
	With additional controls											
Response measure × Covid-19 pandemic	-0.149** (0.043)	-0.041 (0.026)	-0.107** (0.034)	-0.038# (0.020)	-0.010 (0.009)	-0.101** (0.035)	0.080 (0.080)	-0.033 (0.049)	0.113# (0.059)	-0.018 (0.042)	-0.005 (0.009)	0.103 (0.061)
b) Effect by Covid-19 wave	(1)	(2)	(3)	(4)	(5)	(6)	(7)	(8)	(9)	(10)	(11)	(12)
	Baseline											
Response measure × First wave Covid-19	-0.085 (0.088)	0.005 (0.013)	-0.091 (0.079)	0.001 (0.011)	-0.008 (0.008)	-0.077 (0.081)	0.243** (0.071)	0.026 (0.020)	0.217** (0.058)	0.019 (0.018)	0.012 (0.012)	0.213** (0.056)
Response measure × Second wave Covid-19	-0.168** (0.046)	-0.034* (0.015)	-0.134** (0.042)	-0.035* (0.016)	-0.003 (0.012)	-0.130** (0.045)	0.276** (0.080)	0.029 (0.026)	0.247** (0.068)	0.016 (0.024)	0.024* (0.011)	0.237** (0.067)
	With additional controls											
Response measure × First wave Covid-19	-0.123* (0.048)	-0.033# (0.018)	-0.089* (0.042)	-0.033* (0.016)	-0.011 (0.009)	-0.079# (0.044)	0.129# (0.071)	-0.008 (0.028)	0.137# (0.071)	0.004 (0.028)	-0.001 (0.008)	0.126 (0.075)
Response measure × Second wave Covid-19	-0.189** (0.058)	-0.054 (0.040)	-0.135* (0.052)	-0.046 (0.029)	-0.008 (0.009)	-0.136* (0.056)	0.002 (0.113)	-0.074 (0.073)	0.075 (0.068)	-0.054 (0.059)	-0.011 (0.011)	0.067 (0.071)

OLS estimation. The dependent variable is recorded at the top of the column. The unit of observation is a country-month pair. Data cover the period from January 2019 through January 2021 in monthly frequency. Time fixed effects and country fixed effects are included but not reported. Robust standard errors (clustered by time and country) are in parentheses. **, * and # denote significant at the 1%, 5% and 10% level, respectively.

## Appendix

**Table A1:** Correlations Between Government Response Measures to Covid-19

	SC	WC	CPE	RG	CPT	SHR	RIM	ITC	PIC	TP	CT	EIH	IV	FC	VP	PEP	ICS	DCR	FM	INS
School closing	1.00																			
Workplace closing	0.69	1.00																		
Cancel public events	0.74	0.74	1.00																	
Restrictions on gatherings	0.66	0.72	0.80	1.00																
Close public transport	0.54	0.56	0.50	0.49	1.00															
Stay at home requirements	0.64	0.68	0.64	0.64	0.60	1.00														
Restr’s on internal movement	0.62	0.64	0.61	0.55	0.65	0.67	1.00													
International travel controls	0.56	0.50	0.58	0.49	0.41	0.43	0.46	1.00												
Public information campaigns	0.56	0.52	0.62	0.59	0.30	0.43	0.38	0.62	1.00											
Testing policy	0.37	0.37	0.45	0.48	0.17	0.29	0.19	0.41	0.58	1.00										
Contact tracing	0.36	0.32	0.43	0.44	0.15	0.28	0.18	0.39	0.58	0.58	1.00									
Emergency inv’t in healthcare	0.15	0.12	0.11	0.07	0.10	0.08	0.10	0.10	0.08	0.01	0.04	1.00								
Investment in vaccines	0.05	0.06	0.06	0.06	-0.01	0.02	0.06	0.05	0.06	0.08	0.03	0.12	1.00							
Facial coverings	0.37	0.40	0.47	0.53	0.20	0.39	0.32	0.26	0.51	0.49	0.43	-0.07	0.01	1.00						
Vaccination policy	0.09	0.15	0.15	0.19	0.02	0.11	0.04	0.07	0.11	0.21	0.09	-0.02	0.03	0.18	1.00					
Protection of elderly people	0.42	0.46	0.48	0.47	0.31	0.48	0.35	0.32	0.38	0.40	0.32	0.12	0.13	0.26	0.17	1.00				
Income support	0.30	0.35	0.36	0.39	0.09	0.22	0.16	0.34	0.41	0.47	0.42	0.02	0.13	0.24	0.23	0.46	1.00			
Debt/contract relief	0.41	0.42	0.43	0.41	0.24	0.37	0.30	0.39	0.47	0.50	0.43	-0.02	0.04	0.41	0.11	0.37	0.46	1.00		
Fiscal measures	0.18	0.17	0.16	0.11	0.08	0.09	0.14	0.15	0.12	0.02	0.08	0.43	0.15	-0.10	-0.04	0.17	0.09	0.06	1.00	
International support	0.07	0.07	0.07	0.07	0.05	0.05	0.07	0.07	0.05	0.04	0.03	0.19	0.17	-0.05	0.01	0.14	0.09	0.02	0.18	1.00

Observations: 2,335.

## Footnotes

1 Kalemli-Ozcan (2020) notes that the high-frequency real time data on portfolio flows, which are often used as long as official balance of payments data are unavailable, might not be fully informative for emerging markets; see also Koepke and Paetzold (2020).

2 Borgioli et al. (2020) argue, along similar lines, that, after a short period of systemic stress and fragmentation, there has been a fast rebound of European financial integration which has broadly returned to its pre-crisis levels.

3 Since we examine bilateral capital flows of a single country, Germany, our analysis focuses exclusively, by construction, on the impact of government measures imposed by foreign countries, while measures taken by the German government in response to Covid-19 are absorbed by time fixed effects.

4 A detailed description of the SIFCT dataset is available in Biewen and Stahl (2021) and at https://www.bundesbank.de/en/bundesbank/research/rdsc/research-data/statistics-on-international-financial-and-capital-transactions-sifct--831698.

5 Gross bilateral portfolio flows between Germany and the United Kingdom (purchases and sales of foreign securities) have substantially increased in 2020, due to the relocation of the securities business of some Britain-based banks.

6 The dataset and more detailed information are available at https://www.bsg.ox.ac.uk/research/research-projects/coronavirus-government-response-tracker; see Hale et al. (2021).

7 In unreported robustness checks, we have also experimented with translating the information from the indicators into plain binary dummy variables, without much additional insights.

8 Declarants typically report their aggregated positions at the end of the month. There is no further information given about the submission date of the report or the day of the cross-border financial transaction. Also, declarants are, in principle, allowed to report retroactive transactions over the following two months, such that the most recent data may be subject to revisions.

9 Appendix Table A1 presents a correlation matrix, providing further evidence that the indicators of government response measures are not particularly tightly related.

10 Outflows (inflows) are defined as purchase (sale) of foreign assets by German investors and sale (purchase) of German assets by foreign investors.

11 The list of variables used as additional controls includes the nominal effective exchange rate, real effective exchange rate, policy interest rate, the monthly percentage change in the national central bank’s balance sheet, money market rate, long-term bond yield, central bank reserves, consumer price inflation, industrial production, economic policy uncertainty, and the OECD composite leading indicator.

12 For member countries of the euro area, we use the national central bank balance sheets.

13 Bordo (2021) provides a historical account of central bank cooperation during crisis episodes.

14 Put differently, this index drops the economic support measures from the overall government response index.

15 The results remain qualitatively unchanged when we use October 2020 as the start of the second wave.

16 According to the OxCGRT data description, the variable ‘income support’ records “if the government is providing direct cash payments to people who lose their jobs or cannot work”.

17 In 2019, financial transactions in equities accounted for 37% in total financial flows of Germany, followed by bonds (34%), money market instruments (17%), investment certificates (8%), foreign direct investment (2%) and other (2%).

18 As before, the estimate of γ is often statistically insignificant for other measures of government interventions.
